# Therapeutic effects on cancer of the active ingredients in rhizoma paridis

**DOI:** 10.3389/fphar.2023.1095786

**Published:** 2023-02-21

**Authors:** Jie Li, Jinhao Jia, Weiwei Zhu, Jianfei Chen, Qiusheng Zheng, Defang Li

**Affiliations:** ^1^ Collaborative Innovation Platform for Modernization and Industrialization of Regional Characteristic Traditional Chinese Medicine, School of Integrated Traditional Chinese and Western Medicine, Binzhou Medical University, Yantai, Shandong, China; ^2^ Clinical Trial Agency, Yantai Yuhuangding Hospital Affiliated to Qingdao University, Yantai, Shandong, China

**Keywords:** Chinese medicine Rhizoma paridis, Rhizoma paridis saponins, polyphyllin I, polyphyllin II, polyphyllin VI, polyphyllin VII

## Abstract

Cancer is a major threat to human health, with high mortality and a low cure rate, continuously challenging public health worldwide. Extensive clinical application of traditional Chinese medicine (TCM) for patients with poor outcomes of radiotherapy and chemotherapy provides a new direction in anticancer therapy. Anticancer mechanisms of the active ingredients in TCM have also been extensively studied in the medical field. As a type of TCM against cancer, Rhizoma Paridis (Chinese name: Chonglou) has important antitumor effects in clinical application. The main active ingredients of Rhizoma Paridis (e.g., total saponins, polyphyllin I, polyphyllin II, polyphyllin VI, and polyphyllin VII) have shown strong antitumor activities in various cancers, such as breast cancer, lung cancer, colorectal cancer, hepatocellular carcinoma (HCC), and gastric cancer. Rhizoma Paridis also has low concentrations of certain other active ingredients with antitumor effects, such as saponins polyphyllin E, polyphyllin H, *Paris polyphylla*-22, gracillin, and formosanin-C. Many researchers have studied the anticancer mechanism of Rhizoma Paridis and its active ingredients. This review article describes research progress regarding the molecular mechanism and antitumor effects of the active ingredients in Rhizoma Paridis, suggesting that various active ingredients in Rhizoma Paridis may be potentially therapeutic against cancer.

## Research background

With the growth of aging populations globally, the incidence of cancer is also increasing, which seriously affects the quality of life and life expectancy of cancer patients and has become one of the main causes of death in countries worldwide (Torre et al., 2016). Carcinogenesis is a complex process involving multiple causes including cellular damage, inflammation, proliferation, and genomic instability, leading to alterations in several oncogenic pathways that induce cancer (Liao et al., 2019). To date, many procedures are available to treat cancer that some called “local treatments” such as surgery and radiation therapy which are used for specific tumors or areas. And some are “systemic treatments” that affect the entire body, including chemotherapy, immunotherapy, or targeted therapy. In some case, complementary and alternative therapies are also applied in cancer treatment (Chang et al., 2017). However, these approaches are not very effective because of metastasis and recurrence even after surgery and many side effects in patients after radiotherapy and chemotherapy (Long et al., 2015). Hence, it is crucial to develop new and effective anticancer drugs to inhibit tumor growth and improve the quality of life and survival of cancer patients. More and more traditional Chinese medicines (TCMs), such as *Rhizoma Paridis*, toad venom, *Prunella vulgaris*, and *Solanum nigrum*, have been used in anticancer therapy in TCM, which has gradually become an indispensable tool against cancer (Xiang et al., 2019; Zhang et al., 2020; Li et al., 2021; He et al., 2021).

Rhizoma Paridis is the dried root and rhizome of *Paris polyphlla*, a perennial herbaceous plant of the *Liliaceae* (Chinese Pharmacopeia), or *Melanthiaceae* (The World Flora Online) mainly distributed throughout southwest China. Rhizoma Paridis is listed in the *Pharmacopoeia of the People’s Republic of China* as the main TCM for heat-clearing and detoxifying (Commission, 2015). According to TCM theory, Rhizoma Paridis has the above functions as well as those of alleviating inflammation (swelling) and relieving pain; it has been extensively used to treat pharyngitis, venomous snake bites, pain, and convulsions (Ding et al., 2021). Rhizoma Paridis is also the main ingredient in many renowned traditional Chinese patent medicines (TCPMs), such as Yunnan Baiyao and Gongxuening capsules (Cunningham et al., 2018). In addition, Rhizoma Paridis has strong antitumor effects and has been widely used in TCM prescriptions for cancer treatments in recent years.

With the development of chemical extraction technology, the active ingredients of Rhizoma Paridis have gradually been identified. The chemical components of Rhizoma Paridis mainly include steroidal saponins, C21 steroids, flavonoids, polysaccharides, and amino acids (Su et al., 2022). As the main active ingredients, steroidal saponins have been shown to have good pharmacological activity (Qin et al., 2016). Specifically, polyphyllin I, polyphyllin II, polyphyllin VI, and polyphyllin VII have been extensively reported as the main active ingredients against cancer (Wu et al., 2012). In addition, the anti-tumor effects of some low-abundance active ingredients in Rhizoma Paridis have been reported in previous studies, such as Paris polyphylla-22 (PP-22, promotes autophagy and apoptosis in nasopharyngeal carcinoma cells) (Tan et al., 2019), polyphyllin E (inhibits proliferation, migration and invasion in ovarian cancer cells) (Liu. et al., 2022), Paris saponin H (induces cell apoptosis, suppresses EMT and invasion in liver cancer cells) (Chen et al., 2019), formosanin C (inhibits pulmonary metastasis on mouse lung adenocarcinoma) (Man et al., 2011b), and gracillin (induces apoptosis and inhibits migration in BGC823 cells) (Liu et al., 2021). In additional, previous studies have demonstrated anticancer activities of polyphyllin in Rhizoma Paridis in a variety of cancers, including lung cancer (Shuli et al., 2011), gastric cancer (Sun et al., 2007), colon cancer (Teng et al., 2015), prostate cancer (Zhang et al., 2018), and melanoma (Man et al., 2011a). Although increasingly more scientists and experts in the medical field have researched the active ingredients in Rhizoma Paridis, due to its complex combination of active ingredients and anticancer mechanisms (Zhang et al., 2010), further research is needed to reveal the mechanism of some active ingredients in Rhizoma Paridis.

In this article, we reviewed the research results regarding active ingredients in Rhizoma Paridis in regulating tumor cells, and we explore and discuss the mechanisms of the main active ingredients in Rhizoma Paridis in regulating different cancers, different signaling pathways, and different molecular targets.

## Methods and strategies

We searched the published literature the databases Web of Science, PubMed, China National Knowledge Infrastructure, and Wanfang Data up to July 2022 for original research articles related to the antitumor effects of active ingredients in Rhizoma Paridis against cancer and Rhizoma Paridis combination therapy. Key words: cancer, Chinese medicine, Rhizoma Paridis, Rhizoma Paridis saponins, polyphyllin I, polyphyllin II, polyphyllin VI, polyphyllin VII.

### Application of Rhizoma Paridis and research on its active ingredients

Since ancient China, Rhizoma Paridis has generally used in the dosage of 9 g to treat inflammation, infection, sore throat, and bleeding. Pharmacological studies have shown that Rhizoma Paridis has significant antitumor, hemostatic, antiinfection, and antioxidant activities (Zhao et al., 2009; Liu et al., 2012b). Rhizoma Paridis is the main ingredient in well-known TCPMs including Yunnan Baiyao, Gongxuening capsule, and Baibaodan that are mainly used for promoting blood circulation, removing blood stasis, heat-clearing, detoxifying, and regulating menstruation, according to TCM theory (Liu et al., 2009; Yao et al., 2021). Moreover, as a traditional anticancer TCM, the active ingredients of Rhizoma Paridis have been widely studied as potential new anticancer drugs. Studies have shown that the root extract of Rhizoma Paridis has obvious inhibitory effects on lung cancer, gastric cancer, colon cancer, and breast cancer (You et al., 2021). Other studies have also shown that the fruit and aerial parts of Rhizoma Paridis have the effects of inhibiting the migration of cancer cells (Qin et al., 2018; Lin et al., 2021).

**FIGURE 1:**
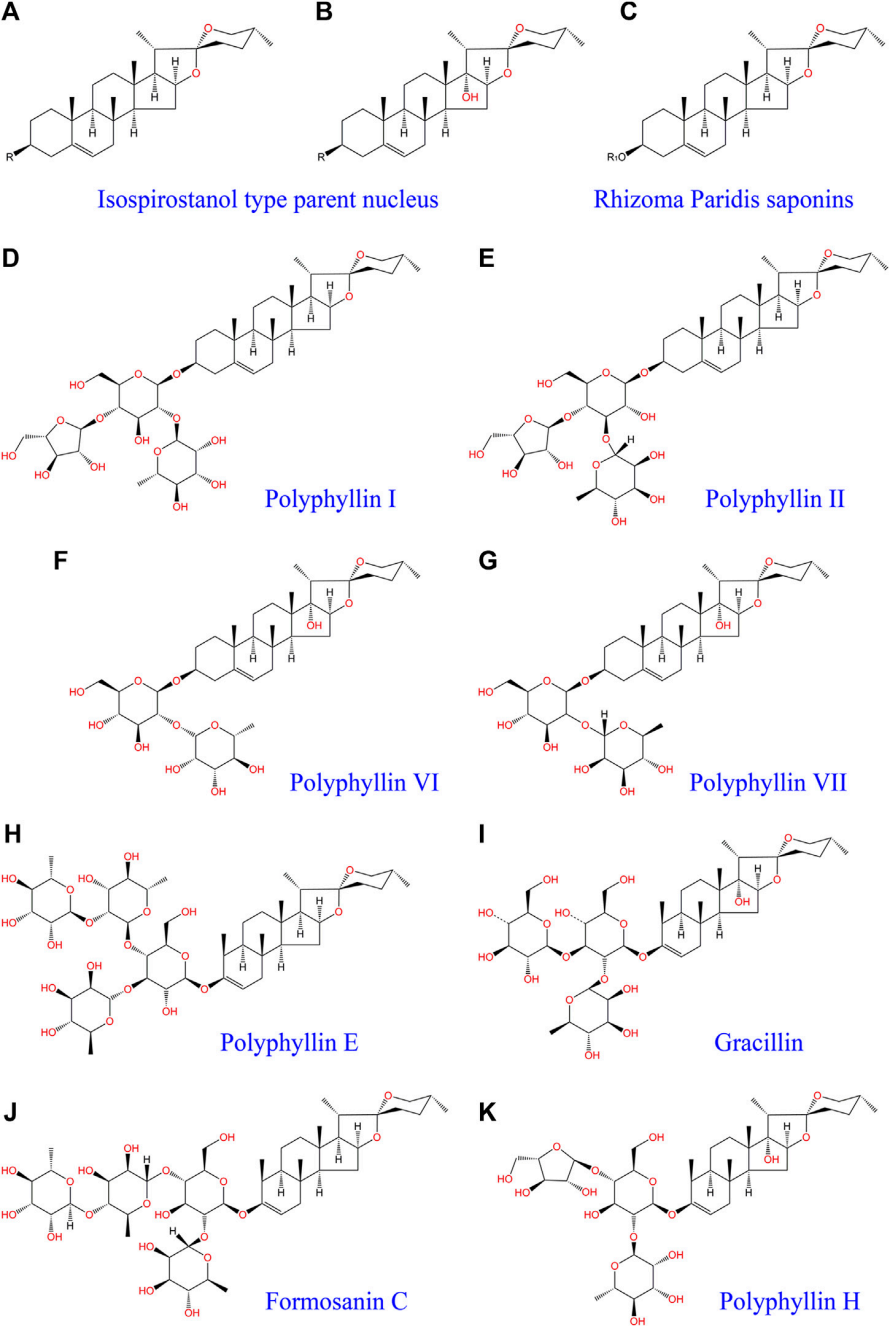
Isospirostanol type parent nucleus **(A,B)**, Rhizoma Paridis saponins **(C)**, polyphyllin I **(D)**, polyphyllin II **(E)**, polyphyllin VI **(F)**, polyphyllin VII **(G)**, polyphyllin E **(H)**, Gracillin **(I)**, Formosanin C **(J)**, and polyphyllin H **(K)**. “R1” indicates that different functional groups represent different types of saponins with different molecular structures.

Research on Rhizoma Paridis extraction and identification has confirmed that the active ingredients in Rhizoma Paridis include steroidal saponins, cholesterol, C21 steroids, phytosterols, insect allergens, triterpenoids, flavonoids, and other chemical compounds (Wei et al., 2014). Steroidal saponins, also known as Rhizoma Paridis saponins (RPS), are the main biologically active chemical components of Rhizoma Paridis and are divided into four types (Kang et al., 2012): spirostanol; isosprirostanol; furostanol; and pseudospirostand and mainly include polyphyllin I, polyphyllin II, polyphyllin VI, formosanin C, polyphyllin VII, gracillin, and other saponin active ingredients (Figure 1). Numerous studies have shown that saponins have antitumor activities in a variety of tumor cells. Among them, five steroidal saponins including total saponins, polyphyllin I, polyphyllin II, polyphyllin VI, and polyphyllin VII have aroused the most attention and have been studied the most. The antitumor activities of these five steroidal saponins have also been verified in a variety of cancers.

### Antitumor effects of RPS

**FIGURE 2:**
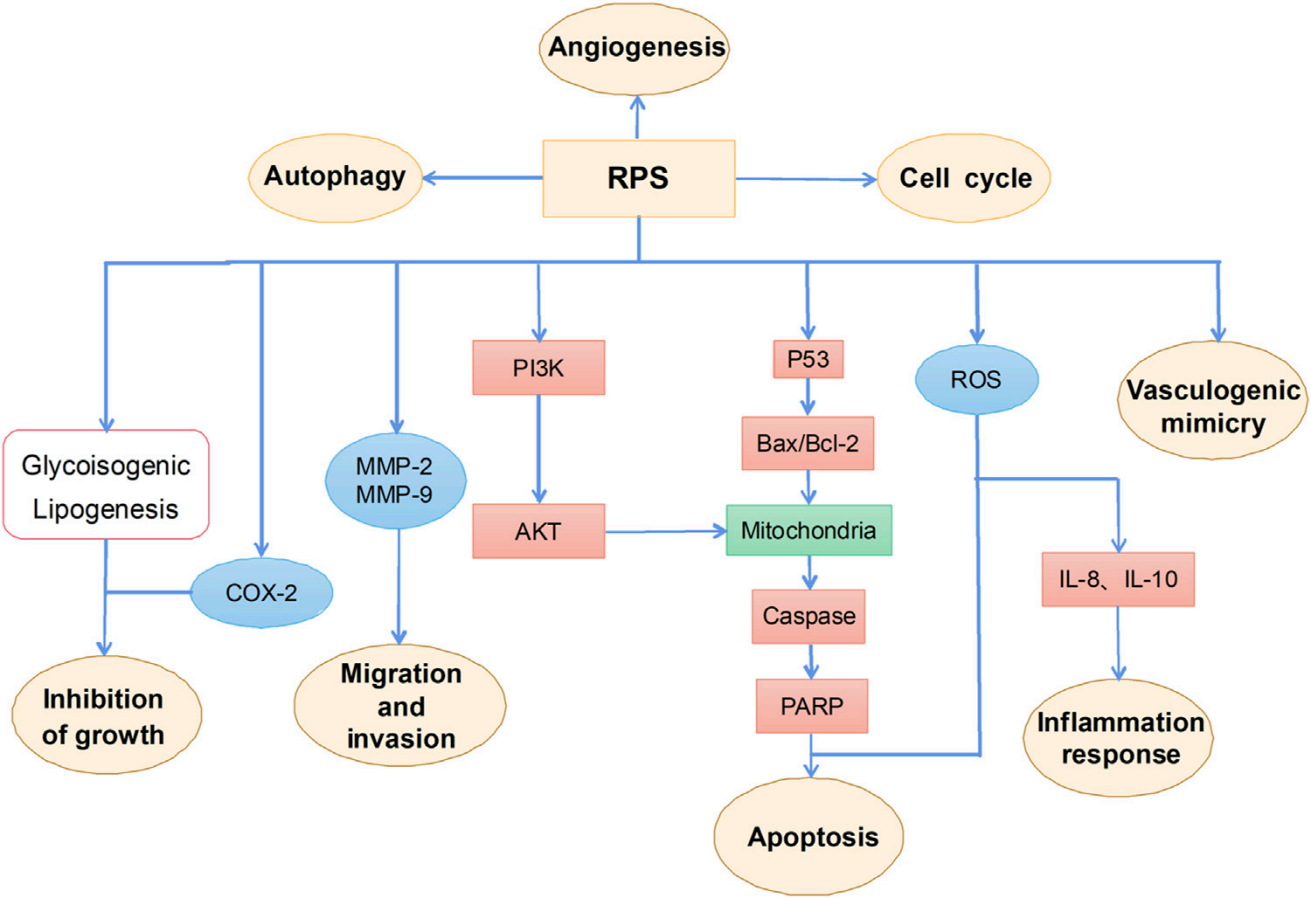
The primary mechanism for the anti-tumor effects of RPS.

RPS is a general term for steroidal saponins in Rhizoma Paridis, with strong activity against hepatocellular carcinoma (HCC), lung cancer, colon cancer, and glioma (Man et al., 2009; He et al., 2014; Qiu et al., 2016; Yao et al., 2018; Lin et al., 2019). RPS has been widely reported as a potential anticancer drug with the main mechanism of inhibiting cell proliferation, inducing apoptosis, autophagy, and cell cycle arrest, as well as enhancing the sensitivity of chemotherapeutic drugs (figure 2).

### Anti-HCC activities of RPS

Protein analysis using matrix-assisted laser desorption/ionization time-of-flight mass spectrometry in RPS-treated hepatoma HepG2 cells revealed that the levels of deoxyuridine triphosphatase, heterogeneous nuclear ribonucleoprotein K, and guanosine monophosphate synthetase were significantly high whereas levels deoxyribonuclease gamma, nucleoside diphosphate kinase A, and centrin-2 were significantly low in the cells. These protein molecules have been confirmed to be closely related to tumorigenesis and tumor progression (Cheng et al., 2008). A recent study showed that RPS treatment in H22 mice bearing HCC not only inhibited but also slowed tumor growth in the mice. RPS also inhibited the metabolism of glycine and serine, reversed aerobic glycolysis, and suppressed adipogenesis, thereby inhibiting the metabolism of tumor cells and effectively suppressing the growth of hepatoma in tumor-bearing mice (Qiu et al., 2016). Another experimental study in H22 tumor-bearing mice also confirmed that RPS significantly reduced levels of glucose, glycine, and alanine and inhibited the fatty acid oxidation pathway and gluconeogenesis pathway that are involved in the body’s energy supply, thereby exerting an anticancer effect (Man et al., 2014b). RPS may also overcome sorafenib resistance in mice hepatocellular carcinoma H22-bearing through the mitochondrial damage pathway and inhibition of phosphatidylinositol-3-kinase (PI3K)/Akt/mammalian target of rapamycin (mTOR) pathway-based lipid synthesis (Yao et al., 2018). In short, RPS is a potential future therapeutic agent against HCC.

### Anti-lung cancer activities of RPS

For a long time, RPS has been studied for its therapeutic effect on lung cancer. Overexpression of matrix metalloproteinases (MMPs) occurs in a variety of malignant tumors and promotes tumor development by promoting cancer cell growth and migration as well as tumor invasion, metastasis, and angiogenesis (Hidalgo and Eckhardt, 2001). RPS inhibits the expression and secretion of MMP2 and MMP9 in A549 lung cancer cells to further inhibit the proliferation, migration, and differentiation of A549 cells (He et al., 2014). Another study has shown that RPS significantly downregulates the expression of P53, B-cell lymphoma 2 (BCL2), and cyclin-dependent kinase 2 (CDK2) and significantly upregulates the expression of P21 and BCL2-associated X (Bax) in A549 cells, thereby inducing cell cycle arrest at the G1 phase. P53 is an important tumor suppressor regulating cell growth and apoptosis in the process of carcinogenesis. RPS directly or indirectly affects the expression of target-regulated p53, thereby changing the expression of downregulated genes including *P21*, *Bax*, *BCL2*, and *CDK2* and resulting in inhibition of cell proliferation, cell cycle arrest, and apoptosis (Zhang et al., 2015). In addition, RPS reduces the level of inflammatory cytokines, tumor necrosis factor-alpha (TNF-α), interleukin (IL)-8 and IL-10 in the serum of C57BL/6 mice through immunomodulation and induces nuclear changes in A549 cells, such as DNA condensation and chromatin fragmentation, thereby inducing apoptosis in the cells. The inhibitory effect of RPS in lung cancer may be achieved by reducing the inflammatory response and inducing tumor cell apoptosis (Li et al., 2013). RPS also has an inhibitory effect in diethyl nitrosamine (DEN)-induced lung adenoma to significantly reduce energy metabolism and glycine, serine, and threonine metabolism, thereby blocking tumor growth. In addition, RPS significantly reduces the expression of inflammatory cytokines, such as TNF-α, IL-6, cyclooxygenase-2 (COX-2), and prostaglandin E2 in lung cancer tissues, reduces the infiltration of inflammatory cells and liver toxicity caused by DEN (Man et al., 2015; Man et al., 2017). In brief, RPS has potential as a future therapeutic agent that can effectively suppress lung tumors.

### Anti-intestinal cancer activities of RPS

Angiogenesis, the development of new blood vessels from pre-existing blood vessels, is an important factor in tumorigenesis and tumor progression (Potente et al., 2011). Tumor progression relies on angiogenesis for nutrient supply and metastasis. RPS has been shown to have selective cytotoxic effects on colon cancer cells (i.e., Lovo cells) and to exhibit significant antiangiogenic effects. RPS inhibits the proliferation, differentiation, and migration of Lovo cells by suppressing angiogenesis; it also induces apoptosis and cell cycle arrest in Lovo cells (Qian et al., 2012). Another study showed that RPS induces death of the colorectal adenocarcinoma cell line DLD-1 by upregulating autophagy markers without triggering apoptosis dependent on p53 and caspase-3, i.e., RPS inhibits cellular DNA production by inducing autophagy. Combined application of RPS doxorubicin to treat colorectal cancer cells showed a stronger anticancer effect than RPS monotherapy (Lin et al., 2019).

### Anti-glioma activities of RPS

Studies have shown that RPS also has a good anticancer effect on glioblastoma, one of the most aggressive type of cancers and among the hardest to treat. Its frequent recurrence limits the therapeutic effects of drugs against glioblastoma, mainly related to the drug resistance of glioblastoma cells (Mrugala, 2013). Drug resistance in glioblastoma treatment is also exhibited with temozolomide (TMZ), a clinical chemotherapeutic drug with broad-spectrum antitumor activity, and DNA-repair enzyme O^6^-methylguanine-DNA methyltransferase (MGMT) plays a key role in TMZ resistance (Lai et al., 2018). A previous study showed that RPS regulates the expression of MGMT by downregulating the expression of PI3K/AKT and its downstream protein nuclear factor κappa-light-chain-enhancer of activated B cells (NF-κB) p65, thereby inhibiting TMZ resistance and inducing mitochondrial apoptosis of U87R cells; results of Cell Counting Kit-8 colorimetric assay and flow cytometry indicated that RPS significantly inhibited the proliferation of glioblastoma cells and TMZ-resistant glioblastoma U87R cells (Zhang et al., 2020).

### Other antitumor activities of RPS

Studies have shown that COX-2 dysfunction is related to digestive system cancer, and its expression level is correlated with the aggressiveness of tumor progression (Misra and Sharma, 2014). A previous study confirmed that RPS significantly reduced the expression of COX-2 and cyclin D1 in rat esophageal tissue and esophageal cancer cells, thereby inducing apoptosis of esophageal cancer cells and cell cycle G2/M arrest. RPS also significantly reduced the release of prostaglandin E2, a downstream molecule of COX-2 in a dose-dependent manner. These results suggest that RPS inhibits the development of esophageal cancer by promoting apoptosis and cell cycle arrest and inhibiting the COX-2 pathway (Yan et al., 2015). RPS exerts antitumor effects in osteosarcoma by inhibiting tumor cell proliferation, metastasis, and vasculogenic mimicry (Yao et al., 2022). In addition, in an experimental study involving a mouse model of cancer pain, it was found that RPS can increase 5-HT and *β*-EP in the brain by inhibiting inflammatory pain caused by oxidative damage, suggesting that RPS has a good therapeutic effect on cancer pain and provides a new tool in the treatment of cancer (Wang G. et al., 2018).

### Antitumor effects of polyphyllin I

**FIGURE 3:**
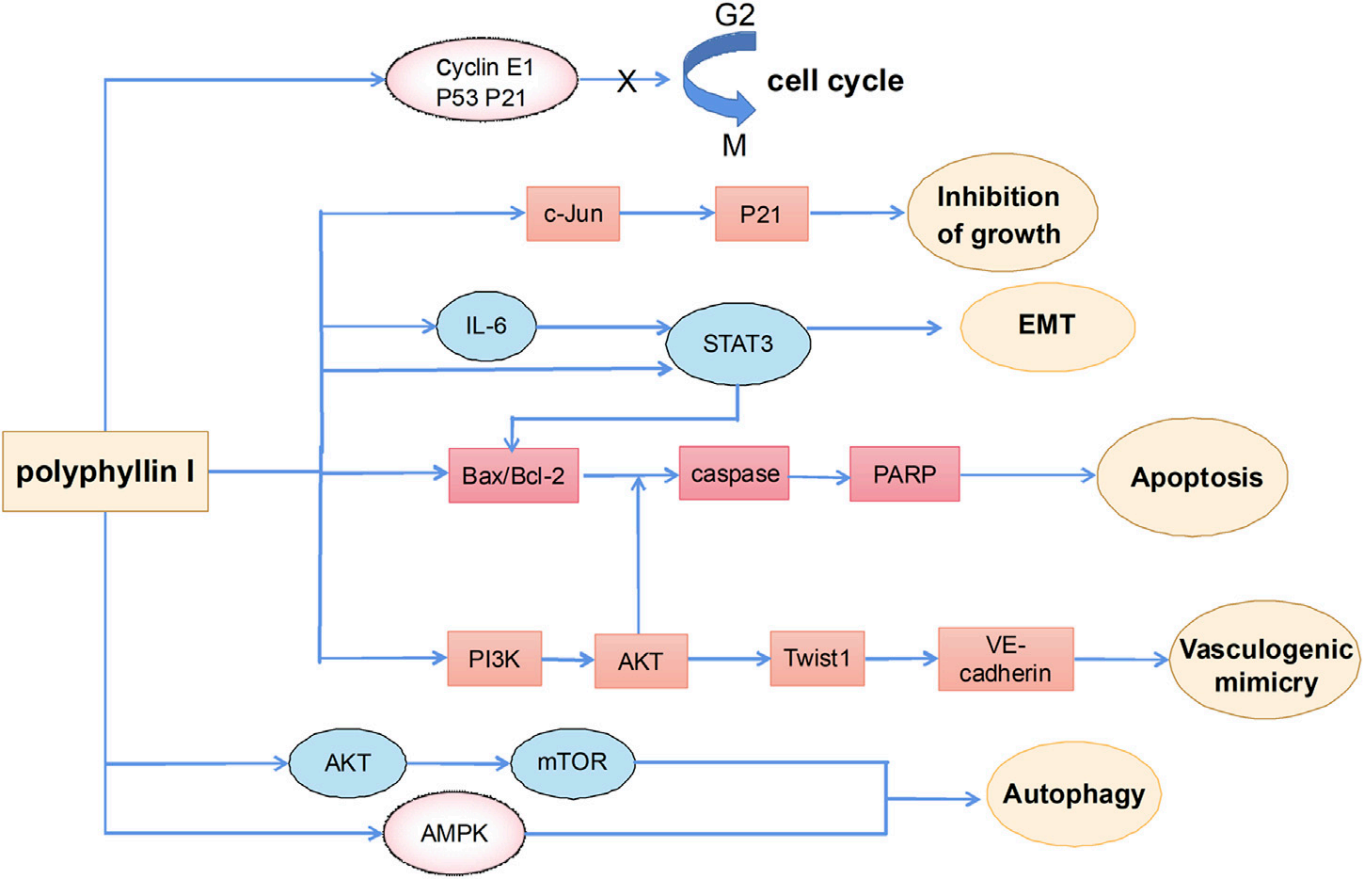
The primary mechanism for the anti-tumor effects of Polyphyllin I.

Polyphyllin I is one of the main active ingredients of steroidal saponins in Rhizoma Paridis and is also known as polyphyllin -D (Li et al., 2001; Lee et al., 2005; Tian et al., 2020). It has relatively strong antitumor effects on a variety of tumors, such as HCC (Cheung et al., 2005), lung cancer (Siu et al., 2008), prostate cancer (Xiang et al., 2018), breast cancer (Li et al., 2017), and malignant glioma (Liu et al., 2017). Polyphyllin I exerts anticancer effects including induction of apoptosis, autophagy and cycle arrest, and inhibition of cell migration and invasion (Figure 3).

### Anti-HCC activities of polyphyllin I

One study showed that after treating HepG2 cells with polyphyllin I, protein levels of p21 and cyclin E were significantly increased whereas the expression of CDK2 and cyclin A2 was significantly decreased, thereby arresting the cell cycle in the G2/M phase and inhibiting tumor cells. In addition, polyphyllin I induced the generation of reactive oxygen species (ROS) and depolarization of MMPs in HepG2 cells, thereby increasing the release of mitochondrial cytochrome c, the Bax/BCL2 ratio, and the activation of caspase-3, caspase-8, and caspase-9, leading to apoptosis of the tumor cells (Zeng et al., 2020). Another study suggested that polyphyllin I inhibited both the formation of angiogenic mimicry by blocking the PI3K-Akt-Twist1-VE-cadherin pathway and transcriptional activation of the Twist1 promoter and interfered with the binding of Twist1 to the VE-cadherin promoter to block the formation of vasculogenic mimicry, thereby inhibiting the metastasis of various cancer cells, including HCC (Xiao et al., 2018). In addition, an animal study confirmed the therapeutic effect of polyphyllin I against HCC and showed that polyphyllin I inhibited HCC by activating caspase-dependent and caspase-independent apoptosis pathways and inhibiting the PI3K/Akt signaling pathway, thereby suppressing tumor growth in a xenograft mouse model injected with HepG2 cells (Chen et al., 2014). The results of the above studies suggest that polyphyllin I has strong potential as an HCC treatment.

### Anti-lung cancer activities of polyphyllin I

Lung cancer, especially non-small cell lung cancer (NSCLC), is considered to be the leading cause of death worldwide and the most prevalent cancer (Siegel et al., 2020). The therapeutic effect of polyphyllin I on lung cancer has been verified in numerous studies. HOX transcript antisense RNA (HOTAIR) is considered to be a potential biomarker for patients with NSCLC that is associated with tumor metastasis and poor prognosis and is highly expressed in NSCLC (Yang et al., 2018). Polyphyllin I reduces the expression of HOTAIR, increases the protein level of the transcription factor c-Jun, and also induces the protein expression and promoter activity of cyclin-dependent kinase inhibitor p21, thereby inhibiting human lung cancer cell growth, migration, and differentiation (Zhao et al., 2019). Another study showed that polyphyllin I increased phosphorylation of stress-induced stimulation of stress-activated protein kinase (SAPK)/c-Jun N-terminal kinase (JNK) and decreased the expression of P65 and DNMT1 protein. Reduction of P65 and DNMT1 protein expression resulted in decreased expression of enhancer of Zeste 2 polycomb repressive complex 2 subunit gene (*EZH2*). As genes that are highly expressed in various cancers, regulating the expression of *DNMT1* and *EZH2* may become novel targets in anticancer therapy (Lin and Wang, 2014). The results of the above studies have clarified that polyphyllin I inhibits the proliferation and differentiation of lung cancer cells by activating SAPK/JNK, reducing the expression of p65 and DNMT1, and inhibiting the expression of *EZH2* (Yu et al., 2016).

Numerous studies have shown that adenosine monophosphate-activated protein kinase (AMPK) is a key energy-sensitive kinase that is widely involved in autophagy (Shi et al., 2012). Polyphyllin I directly binds to allosteric drugs and metabolite sites of AMPK, induces autophagy through AMPK/mTOR signaling, and inhibits the growth of NSCLC cells (Wu et al., 2020). Moreover, after polyphyllin I treatment, the expression of BCL2 in NSCLC cells is reduced, and the expression of Bax and caspase 3 is increased, thereby inducing apoptosis of lung cancer cells (Jiang et al., 2014). Overcoming drug resistance in lung cancer cells is an important research topic. Polyphyllin I reverses resistance of NSCLC cells to osimertinib by regulating the PI3K/Akt signaling pathway (Lai et al., 2021) and overcomes epithelial mesenchymal transition (EMT)-associated resistance to erlotinib in lung cancer cells by inhibiting the IL-6/STAT3 pathway (Lou et al., 2017). Polyphyllin I also induces apoptosis of gefitinib-resistant NSCLC cells by regulating the MALAT1/STAT3 signaling pathway (Yang Q. et al., 2018) and enhances chemosensitivity of cisplatin-resistant NSCLC cells by inhibiting the cellular inhibitor of the protein phosphatase 2 A (CIP2A)/Akt/mTOR signaling axis (Feng F. et al., 2019; Feng F. F. et al., 2019). Interestingly, polyphyllin I combined with hyperthermia arrests the cell cycle in the G2/M phase and promotes apoptosis by regulating the expression of BCL2, Bax, and Caspase-3, ultimately inhibiting the proliferation of NSCLC cells (Zhao et al., 2015). In short, polyphyllin I is expected to be useful in anti-lung cancer therapy.

### Other antitumor activities of polyphyllin I

Many studies have shown the anti-gastric cancer effects of polyphyllin I. For example, application of inhibitors targeting the Janus kinase 2/signal transducer and activator of transcription 3 (JAK/STAT3) pathway (e.g., AG490) may have a strong effect in anti-gastric cancer therapy (Bogani et al., 2013). Studies have confirmed that polyphyllin I mainly inhibits the phosphorylation of STAT3 in a way that inhibits the expression of apoptosis-related protein, such as BCL2, and induces apoptosis of gastric cancer cells (Han et al., 2020). Another experimental study confirmed that polyphyllin I promotes the transformation of mesenchymal cells to epithelial cells, thereby partially inhibiting the migration and invasion of gastric cancer cells. In addition, polyphyllin I downregulates expression of the cellular inhibitor of the *CIP2A* gene and induces degradation of CIP2A protein, downregulation of Akt phosphorylation, and apoptosis of gastric cancer cells. Knocking out of *CIP2A* shows the same effect as polyphyllin I, which also indirectly supports the above findings (Zhang Y. et al., 2018). According to another experimental study, polyphyllin I acts as an inhibitor of pyruvate dehydrogenase kinase 1/Akt/mTOR signaling by promoting the conversion of microtubule-associated protein 1A/1B-light chain 3 (LC3)-I to LC3-II and downregulating cyclin B1 to induce autophagy and cell cycle arrest in gastric cancer HGC-27 cells (He et al., 2019). This also provides an important basis for the anti-gastric cancer effect of polyphyllin I. Moreover, studies have shown that polyphyllin I inhibits prostate cancer invasion and induces apoptosis of prostate cancer cells through the CIP2A/protein phosphatase 2A (polyphyllin 2A)/extracellular signal-regulated kinase (ERK) signaling pathway (Liu X. et al., 2018); it also has a certain inhibitory effect on ovarian cancer cells in mice (Gu et al., 2016).

### Antitumor effects of polyphyllin II

**FIGURE 4:**
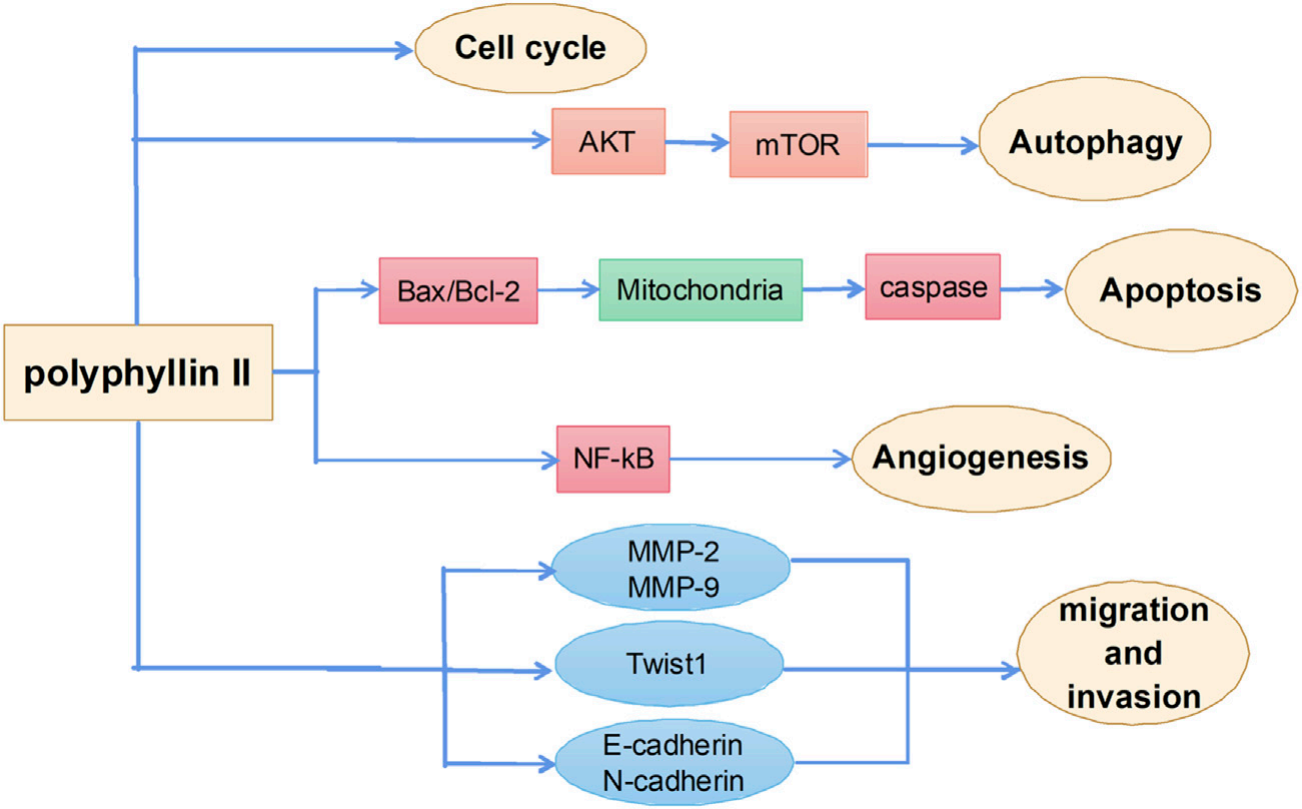
The primary mechanism for the anti-tumor effects of Polyphyllin II.

Polyphyllin II is one of the main active ingredients of steroidal saponins in Rhizoma Paridis and has strong antitumor activities. The main mechanisms by which polyphyllin II exerts antitumor effects include induction of apoptosis, autophagy, and cell cycle arrest and inhibition of metastasis (Figure 4). However, few studies report the effects of polyphyllin II and are mainly all on ovarian cancer. Angiogenesis refers to the growth of new blood vessels from pre-existing vascular endothelial cells. Pathological angiogenesis is best known for its role in gynecological tumor growth and metastasis (Gómez-Raposo et al., 2009). Polyphyllin II inhibits vascular endothelial growth factor (VEGF)-induced phosphorylation of various intracellular proangiogenic kinases, such as extracellular signal-related kinases, AKT kinases, focal adhesion kinases, and Src family kinases, by blocking the activation of VEGF receptor 2 in endothelial cells, thereby inhibiting angiogenesis in the ovarian cancer mouse model and achieving an inhibitory effect on the growth of ovarian cancers. These results suggest that polyphyllin II treatment inhibits almost all essential elements of angiogenesis (i.e., VEGF-induced endothelial cell proliferation, migration, and angiogenesis), with good potential therapeutic importance (Xiao et al., 2014). There is also evidence that polyphyllin II inhibits ovarian cancer cell angiogenesis by regulating NF-κB signaling, thereby inhibiting ovarian cancer cell growth (Yang et al., 2015). Polyphyllin II also increases the expression of pro-apoptotic elements, Bax, cytoplasmic cytochrome c, activated caspase-3, and activated caspase-9 in polyphyllin II-treated SKOV3 cells and reduces the phosphorylation of ERK1/2 and the expression of antiapoptotic BCL2, resulting in cell cycle arrest and apoptosis in ovarian cancer cells (Xiao et al., 2012).

Polyphyllin II also has a good therapeutic effect on HCC by inducing cell cycle arrest and apoptosis through the mitochondrial pathway (Long et al., 2015; Wang et al., 2019b). Experimental data have shown that polyphyllin II also inhibits invasion and metastasis in human bladder cancer by regulating the expression of EMT-related factors and MMPs. Snail family transcriptional repressor 2 (SNAI2) and Twist1 are major transcription factors regulating EMT that may promote tumor metastasis by enhancing cell invasion (Kudo-Saito et al., 2009). A study showed that the expression of SNAI2, Twist1, MMP-2, and MMP-9 in bladder cancer cells was significantly decreased, and cell scratch experiments showed significant inhibition of the migration and invasion of bladder cancer cells after polyphyllin II treatment (Niu et al., 2020). Polyphyllin II also induces autophagy through the Akt/mTOR signaling pathway, thereby promoting apoptosis of breast cancer cells (Xie et al., 2017). In addition, polyphyllin II treatment enhances the sensitivity of lung cancer cells to cisplatin, which provides important data support for polyphyllin II as a chemosensitizer (Man et al., 2020).

### Antitumor effects of polyphyllin VI

**FIGURE 5:**
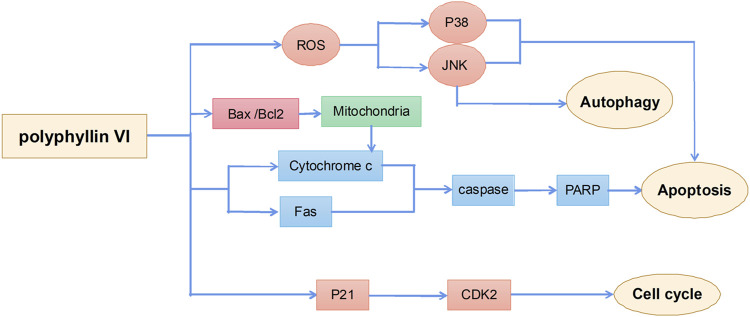
The primary mechanism for the anti-tumor effects of Polyphyllin VI.

Polyphyllin VI is a saponin active ingredient extracted from the root of Rhizoma Paridis and its main anticancer mechanisms include induction of apoptosis, autophagy, and cell cycle arrest (Figure 5). A previous study suggested that polyphyllin VI inhibited glioma growth by increasing the accumulation of ROS in glioma cells and activating the ROS-regulated JNK and P38 pathway, thereby inducing apoptosis and autophagy in cells; applications of the ROS inhibitor N-acetylcysteine significantly attenuated polyphyllin VI-mediated apoptosis and autophagy. These results confirmed that the antitumor activity of polyphyllin VI in glioma cells was *via* the accumulation of ROS and activation of the JNK and P38 pathway (Liu et al., 2020). Polyphyllin VI also effectively inhibited the proliferation of osteosarcoma cells by regulating ROS/JNK activities, blocking the human osteosarcoma cell cycle in the G2/M phase, and inducing apoptosis and autophagy (Yuan et al., 2019). In addition, polyphyllin VI exhibited a strong anti-metastatic effect in a breast cancer 4T1 mouse model by significantly inhibiting cancer cell migration and invasion (Wang P. et al., 2019). Polyphyllin VI also significantly inhibited the proliferation of A549 and NCI-H1299 lung cancer cells by inducing cell cycle arrest in the G2/M phase and apoptosis (Lin et al., 2015).

Unlike other compounds, polyphyllin VI has significant hepatotoxicity. Evaluation of drug hepatotoxicity in HepaRG liver stem cells showed that polyphyllin VI promoted ROS production to induce the release of cytochrome c from mitochondria to the cytoplasm and to activate Fas, caspase-3, caspase-8, caspase-9, and poly (ADP-ribose) polymerase proteins, thereby leading to morphological changes in HepaRG cells and inducing apoptosis. These results suggest that safety evaluation of polyphyllin VI is necessary (Liu Y. et al., 2018).

### Antitumor effects of polyphyllin VII

**FIGURE 6:**
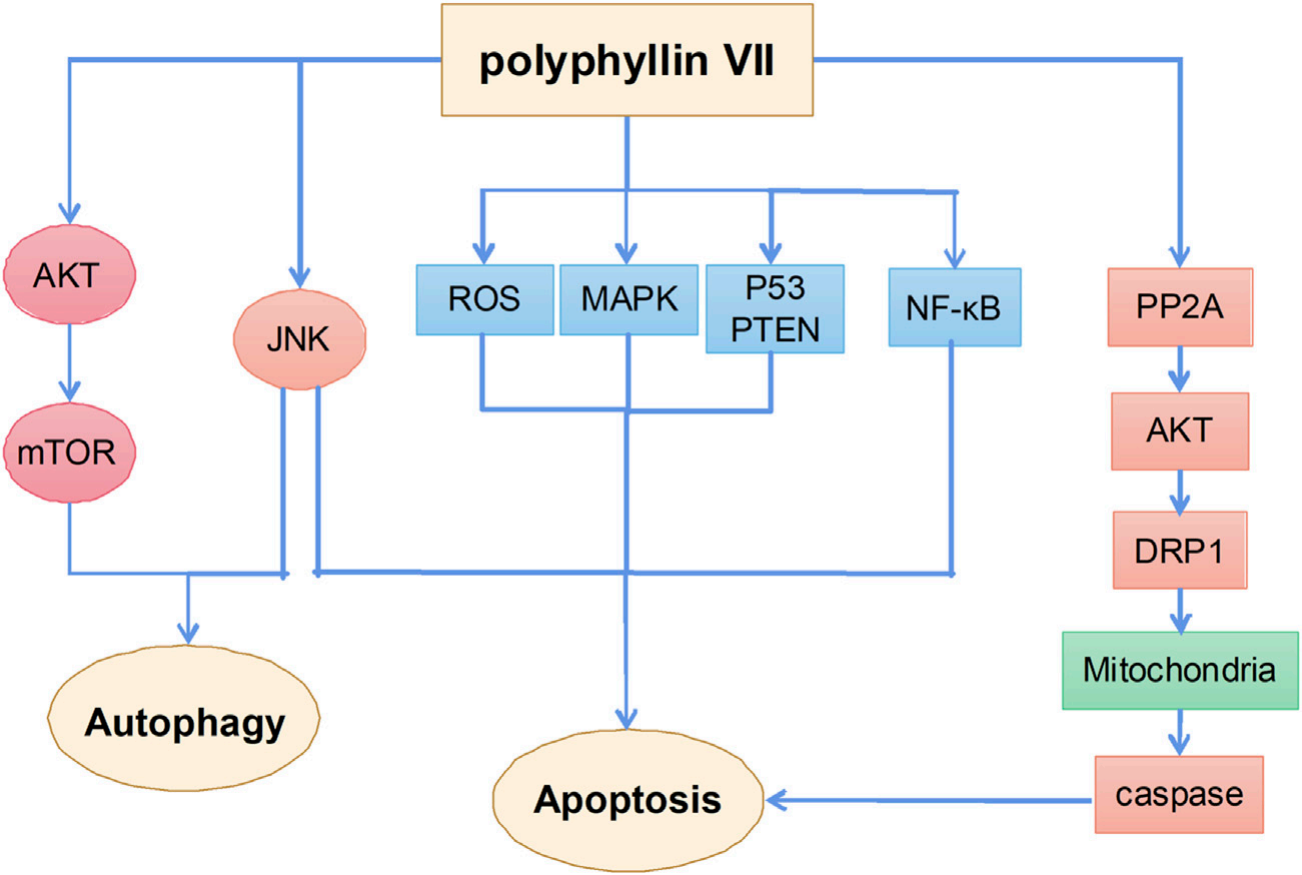
The primary mechanism for the anti-tumor effects of Polyphyllin VII.

Polyphyllin VII is one of the main monomer components of polyphyllin steroid saponins and its anticancer mechanisms mainly include the induction of apoptosis and autophagy (Figure 6). A previous study showed that polyphyllin VII significantly increased the phosphorylation of AMPK and BCL2 and inhibited the phosphorylation of PI3K, AKT, and mTOR in HepG2 cells, thereby inducing autophagy and apoptosis; SP600125 JNK inhibitor pretreatment reversed polyphyllin VII-induced autophagy and apoptosis. These results suggest that polyphyllin VII may induce autophagic cell death in HepG2 cells by inhibiting the PI3K/AKT/mTOR and activating the JNK pathways (Zhang et al., 2016b). Moreover, polyphyllin VII induced apoptosis and autophagy in human osteosarcoma U2OS cells by regulating the JNK pathway (Li X. et al., 2021). Another study showed that polyphyllin VII promoted ROS production in HepG2 cells, leading to depolarization of mitochondrial membrane potential, upregulation of the Bax/BCL2 ratio and protein levels in cleaved forms of caspase-3, caspase-8, and caspase-9, and poly (ADP ribose) polymerase, which eventually led to apoptosis. Polyphyllin VII also significantly enhanced the expression of P53 and phosphatase and tensin homolog (PTEN) and the phosphorylation levels of JNK, ERK, and P38, suggesting that the MAPK and PTEN/P53 signaling pathways are also involved in polyphyllin VII-induced apoptosis of HepG2 cells (Zhang et al., 2016a). Polyphyllin VII also inhibited the growth of human cervical cancer HeLa cells by inducing apoptosis (Zhang et al., 2014).

Notably, polyphyllin VII induces mitochondrial dysfunction not only by promoting reactive oxygen species (ROS) production, but also increasing mitochondrial fission. Dynein-related protein 1 (Drp1) plays an important role in regulating mitochondrial function (Karbowski et al., 2002). Polyphyllin VII enhances the mitochondrial localization of Drp1 by increasing polyphyllin 2A activity and decreasing AKT activity; LB100, a specific polyphyllin 2A inhibitor, attenuates polyphyllin VII-induced mitochondrial fission and apoptosis in SKOV3 ovarian cancer cells. These findings further confirm the role of the polyphyllin 2A/Akt pathway in the regulation of mitochondrial function by polyphyllin VII (Zhao et al., 2021). In addition, polyphyllin VII induces apoptosis in human lung cancer A549 cells by inhibiting the PI3K/Akt and NF-κB pathways, leading to mitochondrial dysfunction (He et al., 2020). Polyphyllin VII also enhances the sensitivity of glioma cells to temozolomide by inhibiting the decreased expression of MGMT (Pang et al., 2019).

### Antitumor effects of other active ingredients in Rhizoma Paridis

In addition to the main active ingredients in Rhizoma Paridis, including RPS, polyphyllin I, and polyphyllin II, some less-abundant active ingredients, such as saponins polyphyllin E, polyphyllin H, *Paris polyphylla*-22, gracillin, and formosanin, also have antitumor effects. According to a previous study, polyphyllin E inhibits ovarian cancer cells by downregulating the AKT/NF-κB pathway, reducing the expression of MMP2 and MMP9, and inhibiting the proliferation, migration, and invasion of ovarian cancer cells, thereby achieving an inhibitory effect in ovarian cancer cells (Liu Y. et al., 2022). In another study, polyphyllin H inhibited the growth of hepatoma cells and xenografts by suppressing the Wnt/β-catenin pathway, while knocking out of the *β*-catenin gene significantly inhibited this phenomenon (Chen T. et al., 2019). In addition, polyphyllin H-treated U251 human glioma cells had upregulated P21 and P27 expression and downregulated cyclin D expression, which further induced cell cycle arrest of U251 cells at the G1 phase. Further experimental data showed that polyphyllin H reduced U251 cell survival by inhibiting the expression of ARA1 and ARA3, subsequently suppressing the phosphorylation of Akt and MAPK, inducing apoptosis and cell cycle arrest, and inhibiting the proliferation of glioma cells (Bi et al., 2021). Another active ingredient, polyphyllin-22, is reported to promote autophagy and apoptosis in CNE-2 nasopharyngeal carcinoma cells by inducing endoplasmic reticulum stress, downregulating the STAT3 signaling pathway, and regulating the MAPK pathway (Tan et al., 2019). In addition, polyphyllin-22 induced cell cycle arrest in S-phase and G2/M-phase and apoptosis in SCC-15 human tongue squamous cell carcinoma cells by activating the p38 and caspase-8/caspase-3 pathways (Ke et al., 2016). Some researchers showed that formosanin C inhibited metastasis of lung adenocarcinoma in mice by inhibiting MMPs through wound healing and migration assays. The anticancer effect of formosanin C is significantly better than that of cisplatin whereas the side effects of formosanin C were fewer (Man et al.). Formosanin C also has an inhibitory effect on HCC cells (Qin et al., 2012; Li et al., 2014). Lastly, a study showed that as a TIPE2 inducer, gracillin inhibits the proliferation and migration of BGC823 gastric cancer cells (Liu et al., 2021).

### The other effects of active ingredients in *Rhizoma Paridis*

Previous studies investigated that besides anti-tumor effects, some active ingredients of *Rhizoma Paridis* have various biological activities such as anti-inflammation, anti-fibrosis, hemostasis, and antibiosis. For instance, polyphyllin I, polyphyllin D, and polyphyllin G were verified have potential anti-inflammatory effects in various inflammatory animal models (Wang Q. et al., 2018; Zhang et al., 2019; Zhu et al., 2019). In additional, CCl4-induced hepatic fibrosis were significantly improved by polyphyllin D, polyphyllin G, polyphyllin VI, and formosanin C (Man et al., 2014a; Hong et al., 2016; Han et al., 2019). The mouse tail snipping model demonstrated that Paris saponin H, polyphyllin I, polyphyllin II, and polyphyllin VII could serve as favorable hemostatic agents (Sun et al., 2014; Wen et al., 2019; Qiao et al., 2021). Polyphyllin V and polyphyllin VII showed powerful antibacterial activity against *propionibacterium acnes* (Qin et al., 2012).

## Outlook for future research

The number of cancer patients increases every year as chemotherapy and radiotherapy fail to address the root cause of cancers. Prolonged administration and increased doses of chemotherapeutic drugs also increase the drug resistance of tumor cells and the possibility of serious side effects in the human body, resulting in treatment failure (Cao et al., 2017). For example, patients are often intolerant to cisplatin-based therapeutic regimens, resulting in poor therapeutic outcomes and the associated adverse reactions, such as hair loss, nausea, and vomiting (Dasari and Tchounwou, 2014). Even after surgical treatments, the chance of metastasis and cancer recurrence is very high (Niibe and Hayakawa, 2010). Under these circumstances, TCM, which has been used for thousands of years, is gradually becoming recognized by oncologists and cancer patients as an option. As an anticancer TCM, Rhizoma Paridis has shown good results in clinical applications for many years. This is of great importance for the scientific and rational application of Rhizoma Paridis and the unique antitumor effects of TCM. Nevertheless, no anticancer drugs use Rhizoma Paridis as the main therapeutic agent in clinical practice. Hence, there is an urgent need to clarify the underlying therapeutic mechanism of the active ingredients in Rhizoma Paridis to lay the foundation for future clinical applications.

Studies have shown that some active ingredients of Rhizoma Paridis exhibit cytotoxicity, such as hepatoxicity. In particular, the saponin component polyphyllin I has relatively strong cytotoxicity. Previous research on the order of cytotoxicity among four saponins in HL-7702 cells showed that polyphyllin I and polyphyllin VII had similar cytotoxicity, followed by polyphyllin II and polyphyllin VI. These four saponins induce apoptosis in liver cells by activating ROS stress and death receptor pathways (Wang et al., 2019c). Animal studies have shown that Rhizoma Paridis causes side effects, such as nausea, vomiting, diarrhea (Liu et al., 2012a), and even hemolysis (Liu J. et al., 2022). Other studies have shown that the water extract of turmeric enhanced the antitumor effects of trichosanthesin and significantly reduced gastric irritation of trichosanthesin, thereby reducing its toxicity (Man et al., 2013; Liu et al., 2014; Man et al., 2016). To comprehensively assess the toxicity of polyphyllin II in the intestinal tract, pharmacologists used the Swiss-rolling technique of intestinal tissue preparation for immunohistochemistry to observe histopathological changes in the entire intestinal tract. The results showed that polyphyllin II had no obvious toxicity at a dose of 20 mg/kg *in vivo* (Chen M. et al., 2019). Intravesical instillation has also been proposed to avoid gastrointestinal toxicity and intravenous incompatibility (Guo et al., 2018). Thus, future research and development of the TCM Rhizoma Paridis should not only focus on enhancing its antitumor activities and clarifying its antitumor mechanism but also on reducing its toxicity and side effects. More clinical studies of the therapeutic safety of Rhizoma Paridis should be carried out to ensure the safety and efficacy of this TCM medicine and its active ingredients in anticancer therapy.

## Conclusion

Many chemotherapeutic drugs cause serious adverse reactions in clinical application, resulting in poor patient outcomes (Islam et al., 2019). There is an urgent need to develop new therapeutic drugs against cancers with high efficacy and less toxicity. Recent evidence has shown that the combination of chemotherapy drugs and TCM has better therapeutic efficacy and reduces the side effects of chemotherapy (Zhang and Xiao, 2021). Under these conditions, anticancer components extracted from natural medicines may become the most promising anticancer drugs. As a representative antitumor drug in TCM, Rhizoma Paridis has a wide range of clinical applications. In this review article, we discussed the antitumor effects and molecular mechanisms of the main active ingredients in Rhizoma Paridis. According to previous literature, the inhibitory effects of these active ingredients on tumor cells are achieved through various ways, such as apoptosis, autophagy, cell cycle arrest, inhibiting metastasis, and reversing drug resistance (Table 1). These findings suggest that active ingredients, such as Rhizoma Paridis saponins, may be potential drugs for the clinical treatment of cancer in the future. However, further experimental studies are needed to elucidate the exact molecular mechanisms prior to its clinical application. In addition, the complex composition of compounds extracted from Rhizoma Paridis and the toxicity of Rhizoma Paridis itself also limit its clinical application. Hence, in-depth research is required to apply the active ingredients extracted from Rhizoma Paridis to clinical settings. This article aims to provide theoretical support and medication guidance for the clinical application of Rhizoma Paridis by summarizing the therapeutic efficacies of the active ingredients of Rhizoma Paridis in anticancer therapy.

**TABLE 1:** The antitumor activities and mechanisms of Rhizoma Paridis saponins.

Compounds	Subjects (cells/animals)	Concentration	Safe dose for animals	Research mechanisms	Main mechanisms	Tumor type	References
Rhizoma Paridis saponins	T739 mice, H22 mice, C57BL/6 mice, U87 cells, A549 cells, Lovo cells, DLD-1 cells, EC9706 cells, and 143B cells	0.1–120 µM	0–200 mg/kg	MMP-2, MMP-9 synthetic signaling pathway, Glycolysis and lipogenesis pathway, Fatty acid oxidation sugar isomerization pathway, ROS/PI3K/Akt pathway, PI3K/Akt/mTOR pathway, p53 signaling pathway, Caspase pathway, Cyclooxygenases-2 Pathway, Vasculogenic Mimicry signaling pathway, Mitochondrial pathway	Apoptosis, cell necrosis, inhibition of proliferation and migration, cell cycle arrest, inhibition of angiogenesis, inhibition of metastasis and invasion, induction of autophagy, inhibition of lipid synthesis	Lung cancer, Pulmonary adenoma, Hepatocellular carcinoma, Glioblastoma, Colorectal cancer, Esophageal cancer and Osteosarcoma	Cheng et al. (2008), Man et al. (2009), Qian et al. (2012), Li et al. (2013), Man et al. (2014b), He et al. (2014), Man et al. (2015), Yan et al. (2015), Zhang et al. (2015), Qiu et al. (2016), Man et al. (2017), Wang et al. (2018a), Yao et al. (2018), Lin et al. (2019), Zhang et al. (2020), and Yao et al. (2022)
Polyphyllin I	MCF-7 cells, HepG2 cells, PC3 cells, MDA-MB-231 cells, U251 cells, A549 cells, PC9 cells, HCC827 cells, SGC7901 cells, PC3 cells, HGC-27cells, and Ovarian cancer metastasis mice	0.1–20 μM	0–5 mg/kg	Mitochondrial pathway, Endoplasmic reticulum stress, Suppression of MUC1 gene expression, Mitochondrial autophagic pathway, JNK signaling pathway, Fas- and mitochondria-mediated pathways, Twist1/VE-cadherin pathway, AMPK/mTOR signaling pathway, Caspase pathway, c-Jun And HOTAIR signaling pathway, SAPK/JNK signaling pathway, PI3K/Akt signaling pathway, IL-6/STAT3 signaling pathway, MALAT1/STAT3 signaling pathway, CIP2A/AKT/mTOR signaling pathway, CIP2A/PP2A/ERK signaling pathway, PDK1/Akt/mTOR signaling pathway, CIP2A/PP2A/AKT signaling pathway	Apoptosis, cell necrosis, inhibition of proliferation and migration, cell cycle arrest, inhibition of angiogenesis, inhibition of metastasis and invasion, induction of autophagy, enhancement of cell sensitivity	Breast cancer, Hepatocellular carcinoma, NSCLC, Prostate cancer, Glioblastoma, Gastric cancer, and Ovarian cancer	Cheung et al. (2005), Siu et al. (2008), Chen et al. (2014), Jiang et al. (2014), Lin and Wang (2014), Zhao et al. (2015), Gu et al. (2016), Yu et al. (2016), Li et al. (2017), Liu et al. (2017), Lou et al. (2017), Liu et al. (2018a), Yang et al. (2018a), Zhang et al. (2018b), Xiang et al. (2018), Xiao et al. (2018), Feng et al. (2019a), Feng et al. (2019b), He et al. (2019), Zhao et al. (2019), Han et al. (2020), Wu et al. (2020), Zeng et al. (2020), and Lai et al. (2021)
Polyphyllin II	SKOV3 cells, HL-7702 cells, HepG2 cells, T24 cells, MCF-7 cells, and NCI-H520 cells	0.1–20 μM	0–25 mg/kg	Regulation of angiogenic factors, ERK signaling pathway and Mitochondrial pathway, NF-κB signaling pathway, Caspase pathway, JNK signaling pathway, MMP-2, MMP-9 synthetic signaling pathway, Akt/mTOR signaling pathway	Apoptosis, cell necrosis, inhibition of proliferation and migration, cell cycle arrest, inhibition of angiogenesis, inhibition of metastasis and invasion, enhancement of cell sensitivity	Ovarian cancer, Hepatocellular carcinoma, Bladder cancer, Breast cancer, and NSCLC	Xiao et al. (2012), Xiao et al. (2014), Long et al. (2015), Yang et al. (2015), Xie et al. (2017), Wang et al. (2019b), Man et al. (2020), Niu et al. (2020)
Polyphyllin VI	U87 cells, U2OScells, 4T1 cells, A549 cells and HepaRG cells	0.1–30 μM	0–5 mg/kg	JNK and P38 signaling pathway, ROS/JNK signaling pathway, Targeted regulation of Rell2, p53 signaling pathway, Fas -pathway and mitochondrial pathway	Apoptosis, cell necrosis, inhibition of proliferation and migration, cell cycle arrest, inhibition of metastasis and invasion	Glioma, Osteosarcoma, Breast cancer, Hepatocellular carcinoma. and Lung cancer	Lin et al. (2015), Liu et al. (2018b), Wang et al. (2019a), Yuan et al. (2019), Liu et al. (2020)
Polyphyllin VII	HepG2 cells, U2OS cells, SKOV3 cells, A549 cells, Hela cells, and U251 cells	0.01–100 µM	0–5 mg/kg	PI3K/AKT/mTOR signaling pathway, JNK signaling pathway, MAPK pathway and P53 signaling pathway, PP2A/AKT/DRP1 signaling pathway, PI3K/Akt and NF-κB signaling pathway, Caspase pathway, AKT signaling pathway	Apoptosis, cell necrosis, inhibition of proliferation and migration, inhibition of metastasis and invasion, induction of autophagy, enhancement of cell sensitivity	Hepatocellular carcinoma, Osteosarcoma, Ovarian cancer, Lung cancer, Cervical cancer, and Glioma	Zhang et al. (2014), Zhang et al. (2016a), Zhang et al. (2016b), Pang et al. (2019), He et al. (2020), Li et al. (2021b), Zhao et al. (2021)
